# The role of local non-tetragonal polar displacements in the temperature- and pressure-induced phase transitions in PbTiO_3_-Bi*Me*O_3_ ferroelectrics

**DOI:** 10.1038/s41598-024-57765-w

**Published:** 2024-03-26

**Authors:** Irina Margaritescu, Zenghui Liu, Zuo-Guang Ye, Boriana Mihailova

**Affiliations:** 1 Department of Earth Sciences, Universität Hamburg, Grindelallee 48, 20146 Hamburg, Germany; 2 Electronic Materials Research Laboratory, Key Laboratory of the Ministry of Education & International Center for Dielectric Research, School of Electronic Science and Engineering, Xi’an Jiaotong University, Xi’an, 710049 China; 3 Department of Chemistry and 4D LABS, Simon Fraser University, Burnaby, BC V5A 1S6 Canada

**Keywords:** Ferroelectrics and multiferroics, Phase transitions and critical phenomena

## Abstract

In situ high-pressure/high-temperature Raman-scattering analyses on PbTiO$_3$, 0.92PbTiO$_3-$0.08Bi(Zn$_{0.5}$Ti$_{0.5}$)O$_3$ and 0.83PbTiO$_3-$0.17Bi(Mg$_{0.5}$Ti$_{0.5}$)O$_3$ single crystals reveal an intensity transfer between the fine-structure components of the A$_1$(TO) soft mode. The enhancement of the lowest-energy subpeak, which stems from intrinsic local non-tetragonal polar distortions, along with the suppression of the tetragonal A$_1$(1TO) fundamental mode with increasing pressure and temperature indicates the key role of the local polarization fluctuations in transformation processes and emphasizes the significance of the order-disorder phenomena in both the pressure- and temperature-induced phase transitions of pure PbTiO$_3$ and its solid solutions with complex perovskites. Moreover, the temperature and pressure evolution of the fraction of the local non-tetragonal polar distortions is highly sensitive to the type of B-site substituent.

## Introduction

Many of the outstanding macroscopic properties observed in ferroelectric materials, including high electromechanical coupling, very high Curie temperatures, enhanced thermal stability, and large coercive fields, are closely linked to local-scale structural phenomena^1–5^. Therefore, a fundamental requirement for engineering novel ferroelectrics capable of withstanding extreme conditions is to understand how the nature of the processes and competing interactions that take place at different length-scales in a material affect its macroscopic properties.

Lead titanate (PbTiO$_3$) is one of the most studied ferroelectric materials. PbTiO$_3$ has a simple, highly polar ABO$_3$ perovskite-type structure and it is an end member of technologically important ferroelectric solid solutions such as PbZr$_{(1-x)}$Ti$_x$O$_3$, $(1-x)$PbTiO$_3-x$Bi*Me*O$_3$ and $(1-x)$PbTiO$_3-x$PbMe$^{2+}_{1/3}$Me$^{5+}_{2/3}$O$_3$^6–11^.

**Figure 1:**
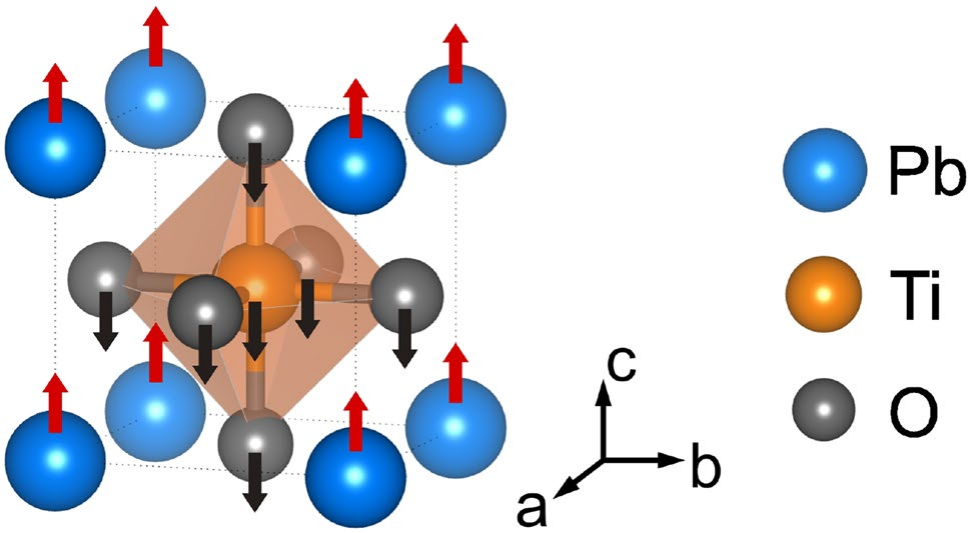
Sketch of the atomic vibrations associated with the optical A$_1$(1TO) mode, plotted using VESTA^25^.

Upon heating, PbTiO$_3$ undergoes a ferroelectric-to-paraelectric phase transition at 763 K from a tetragonal *P*4*mm* to a cubic $Pm\bar{3}m$ phase^6^, whereas two pressure-induced structural phase transitions occur at room temperature: *P*4*mm* ${\mathop {\rightarrow }\limits ^{13 \ \text {GPa}}} R3c {\mathop {\rightarrow }\limits ^{27 \ \text {GPa}}}$ $R\bar{3}c$^12^. For a long time, the temperature-driven phase transition in PbTiO$_3$ had been considered to be a typical soft-mode-driven displacive transition^13^, until several studies revealed a significant contribution of order-disorder processes around the Curie temperature $T_C$^14–18^. Particularly the lowest-energy non-degenerate transverse-optical mode A$_1$(1TO) near 148 cm$^{-1}$, which involves vibrations of the A-site cations against the BO$_6$ octahedra parallel to the direction of the spontaneous polarization^19^ (Figure 1), has attracted significant interest due to its anomalous asymmetric multicomponent Raman-peak shape consisting of four subpeaks^20–24^.

The first model to explain the anomalous peak shape of A$_1$(1TO) in PbTiO$_3$ was proposed by Foster et al.^20,21^, who ascribed the asymmetry of the soft mode to the anharmonic nature of the interatomic potential and assigned the three additional lower-energy subpeaks to the transitions between adjacent excited phonon states in the double-well potential. However, further studies of the Raman scattering at elevated temperatures^22,23^ showed that the suppression of the higher-energy subpeaks (here labeled P$_1$-P$_3$, in agreement with another study^23^) and the enhancement of the lowest-energy subpeak (labeled P$_4$) with increasing temperature were inconsistent with the phonon anharmonicity theory. Hence, a modified model has been proposed by Cho et al.^23^, in which P$_4$ arises from frozen local polarization fluctuations from the direction of the macroscopic polarization, which are confined around thermodynamically unavoidable crystal-lattice defects. These “defect” local polar dipoles have been assumed to have a critical temperature lower than the inherent critical temperature of the matrix, and, as the temperature approaches $T_C$, the correlation between the “defect” polar regions increases and influences the high-temperature behavior of the whole crystal^23^. However, the contribution of these intrinsic non-tetragonal local polar distortion to the pressure-induced phase transitions of PbTiO$_3$ has been neglected, as the Raman scattering near 148 cm$^{-1}$ has been regarded as a single peak to date^26–28^. In addition, studies of the effect of co-substitutions at A and B sites on the subpeak structure of A$_1$(1TO) are scarce^23,24,29^.

Recently, compounds of the complex perovskite PbTiO$_3-$Bi*Me*O$_3$ binary system (*Me* = Sc, In, Zn$_{0.5}$Zr$_{0.5}$, Ni$_{0.5}$Ti$_{0.5}, \ldots$) have attracted significant attention due to their potential applications in high-temperature electromechanical devices^4,30,31^. Of notable technological significance are the (1$-x$)PbTiO$_3-x$Bi(Zn$_{0.5}$Ti$_{0.5}$)O$_3$ and (1$-x$)PbTiO$_3-x$Bi(Mg$_{0.5}$Ti$_{0.5}$)O$_3$ solid solutions, which exhibit stable piezoelectric and mechanical properties at high temperatures^9,32–34^ and show a near-zero volume thermal expansion for low levels of *x*^35^. Furthermore, (1$-x$)PbTiO$_3$-*x*Bi(Zn$_{0.5}$Ti$_{0.5}$)O$_3$ is one of the very few ferroelectric solid solutions which exhibit enhanced tetragonality with increasing *x*^34^.

In order to to elucidate the behavior of the A$_1$(1TO) fine structure near and above $T_C$, we performed temperature-dependent Raman experiments with small temperature increments up to 1000 K on single crystals of PbTiO$_3$ (PT), as well as on two complex PbTiO$_3$-based oxide solid solutions, 0.92PbTiO$_3-$0.08Bi(Zn$_{0.5}$Ti$_{0.5}$)O$_3$ (PT-0.08BZT) and 0.83PbTiO$_3-$0.17Bi(Mg$_{0.5}$Ti$_{0.5}$)O$_3$ (PT-0.17BMT), which undergo a ferroelectric (*P*4*mm*)-to-paraelectric($Pm\bar{3}m$) phase transition at 805 K^34^ and 810 K^9^, respectively. Furthermore, we report the pressure dependence of the A$_1$(1TO)-subpeaks for the same materials up to 10 GPa. Our experimental results show that for all three compounds, the “defect” mode P$_4$ is the dominant excitation that softens upon pressure or temperature increase, that is, the intrinsic non-tetragonal polar fluctuations within the tetragonal polar matrix contribute significantly to both the pressure- and temperature-driven phase transitions. Furthermore, we show that the temperature range of the ongoing processes involving the polarization fluctuations is heavily influenced by the type of chemical bonding between the B$^{2+}$ cation and the oxygen anions.

## Results

**Figure 2:**
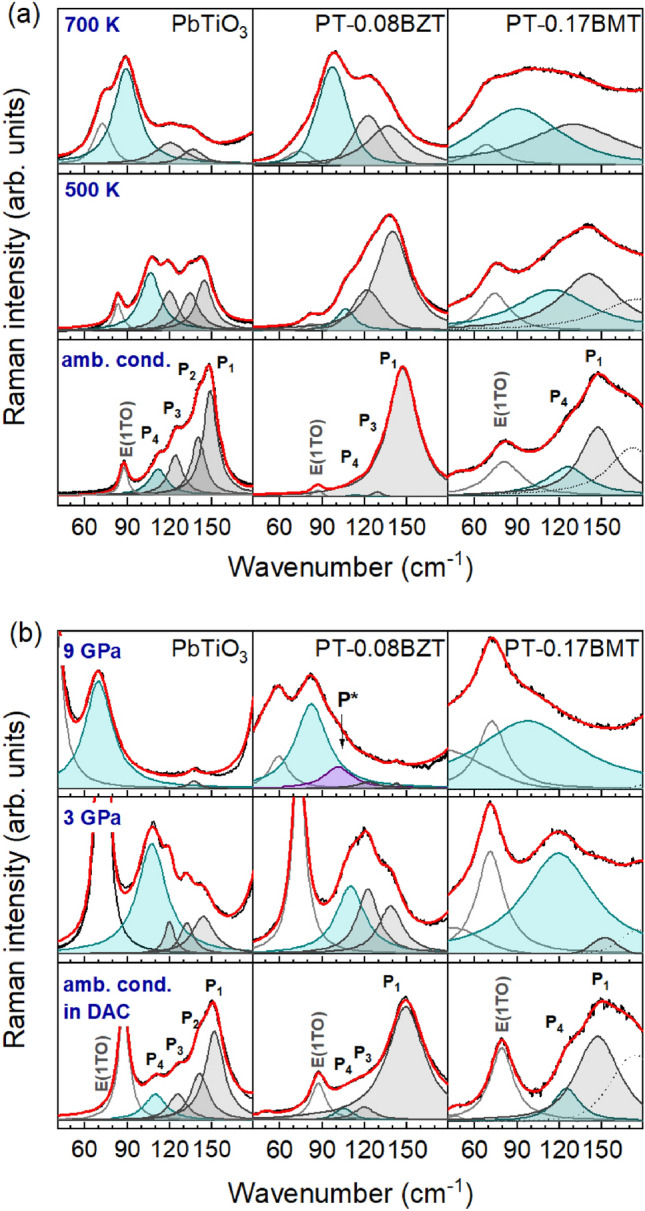
Raman spectra of the A$_1$(1TO) phonon as a function of (**a**) temperature and composition and (**b**) pressure and composition. The fitting peak functions are labeled from P$_1$ to P$_4$, from the highest to the lowest energy. All spectra were collected in the $\bar{x}(yy)x$ backscattering geometry (Porto’s notation^36^, *x* and *y* parallel to the tetragonal [100] and [010] directions, respectively) and were normalized to the intensity of the highest peak. The presence of the E(1TO)-peak in the spectra is related to a slight deviation from the ideal orientation of the single crystals and, in the case of experiments in DAC, the depolarization effects of the diamond anvils.

**Figure 3:**
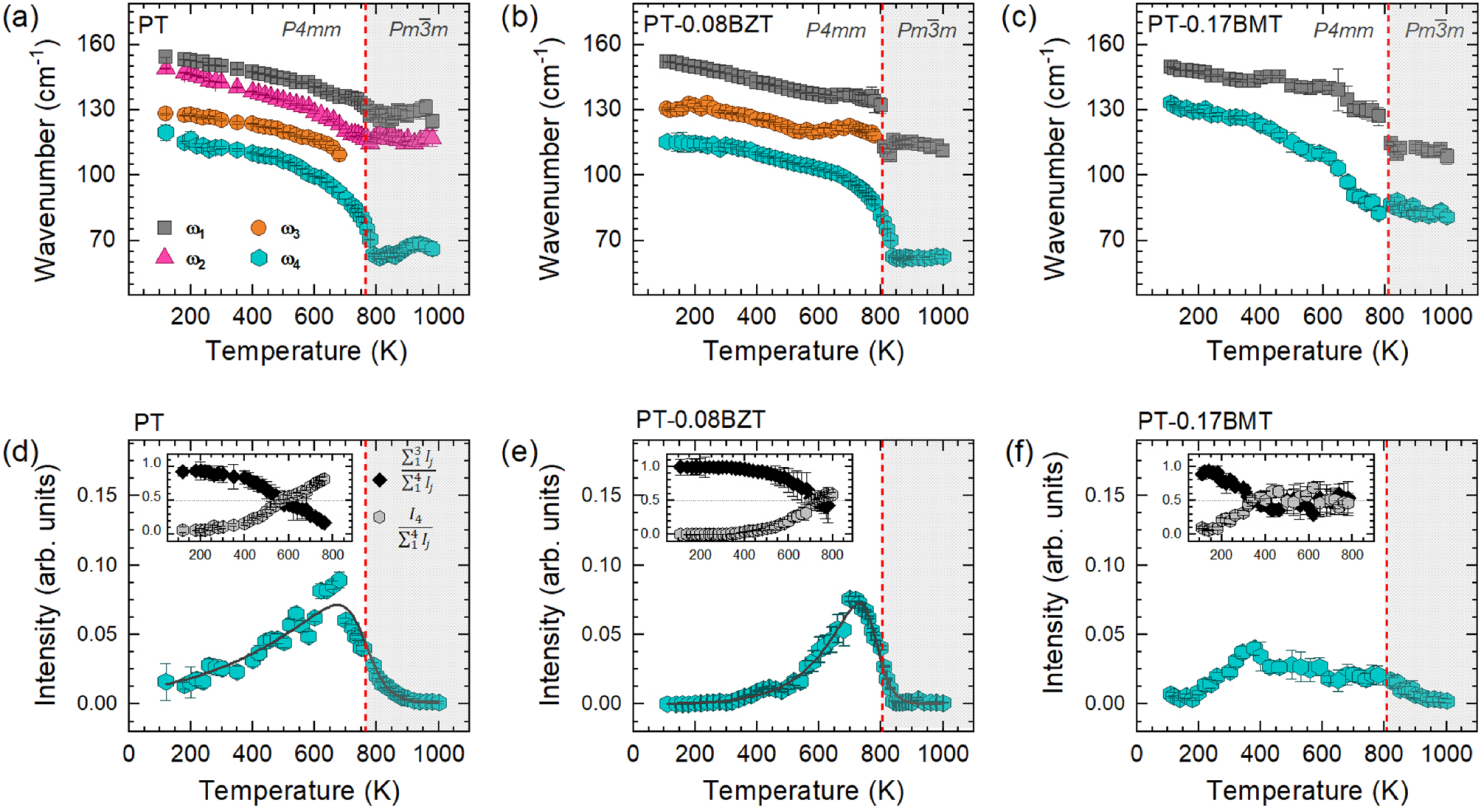
(**a**–**c**) Wavenumber $\omega$ of the A$_1$(1TO) phonon-subpeaks as a function of temperature and composition. The red dashed lines mark the Curie temperature of each compound. (**d**–**f**) Raman intensity $I_4$ of the lowest-energy peak as a function of temperature and composition. $I_4$ of each compound was normalized to the total integrated intensity of the Raman spectrum measured at 300 K. $I_4^{PT}$ and $I_4^{PT-0.08BZT}$ were fitted with an asymmetric double Sigmoidal function. The insets show the intensity ratios $\frac{I_1+I_2+I_3}{I_1+I_2+I_3+I_4}$ (black symbols) and $\frac{I_4}{I_1+I_2+I_3+I_4}$ (gray symbols).

Figure 2a and b show the A$_1$(1TO)-related Raman scattering of PT, PT-0.08BZT and PT-0.17BMT single crystals at selected temperatures and pressures, respectively. The entire Raman spectrum of PbTiO$_3$ measured under ambient conditions is presented in Fig. S2 (Supplementary Material). The anomalous line shape of A$_1$(1TO) can be observed for all three compounds, but all four previously reported subpeaks^20–22^ could only be resolved for pure PT. Due to the substitution disorder, an increase in the width of the Raman peaks is observed for PT-0.08BZT and PT-0.17BMT. Consequently, for PT-0.08BZT the anharmonicity-related subpeak P$_2$ could not be resolved from the fundamental A$_1$(1TO) (peak P$_1$), whereas for PT-0.17BMT none of anharmonicity-related subpeaks P$_2$ and P$_3$ can be resolved. However, for both PT-0.08BZT and PT-0.17BMT the defect-related, non-tetragonal subpeak P$_4$ can be separated from the Raman scattering related to the A$_1$(1TO) mode of the tetragonal matrix and the relative errors of the fitted spectral parameters are within the commonly accepted mathematical criteria (see also Supplementary Material, Fig. S3, and Fig. S4). The suppression of the fundamental phonon mode P$_1$ and the two subpeaks related to the anharmonicity of the interatomic potential (P$_2$ and P$_3$), as well as the enhancement of the “defect” peak P$_4$ upon temperature and pressure increase, can be observed for all samples. Figure 3a–f show the temperature dependences of the wavenumber $\omega$ of all subpeaks as well as the Raman intensity $I_4$ of the lowest-energy subpeak for PT, PT-0.08BZT, and PT-0.17BMT, respectively. As in other perovskite-type ferroelectric materials, forbidden Raman scattering is observed above the Curie temperatures of all three compounds, revealing the existence of local polar distortions within the cubic matrix^15,18,37–39^.

As can be seen in Fig. 3a, for PT $\omega _4$(T) has a much more pronounced non-linear character than $\omega _1(T)$, $\omega _2(T)$ and $\omega _3(T)$. A power function $\omega (T) \propto (T_C - T)^{n}$ was used to fit $\omega _4(T)$, with $\omega _{4}^{PT}(T) \propto (765 - T)^{0.40\pm 0.03}$. The value of *n* $\approx$ 0.40 indicates that the thermal evolution of non-tetragonal local defects is relatively gradual, exhibiting second-order-transition behavior^40,41^, although the PT matrix undergoes a first-order phase transition as revealed by the fundamental E(1TO) phonon mode and macroscopic spontaneous polarization^13,42^. One can speculate that upon heating, the “defect” P$_4$ mode, which behaves as a non-degenerate A-type excitation, disturbs the correlation length of the tetragonal distortion along the direction of the macroscopic spontaneous polarization, while the softening of the fundamental doubly degenerated E(1TO) mode^13,18^ ensures the destabilization of the correlated tetragonal distortions within the plane perpendicular to the direction of macroscopic polarization. As a result, above $T_C$ an ergodic state of dynamic local ferroic distortions is established, which only on average appears as having a primitive cubic symmetry^17,18^.

The gradual suppression of the fundamental mode P$_1$ and the anharmonic subpeaks P$_2$ and P$_3$ upon heating observed in Fig. 2a is a consequence of the reduction of the magnitude of polar tetragonal distortions while approaching $T_C$ from below^17^. In contrast, $I_4(T)$ exhibits an increase up to $\sim$680 K, followed by a decrease (Fig. 3d). However, the full width at half maximum (FWHM) of P$_4$ does not show any anomalous increase near 680 K (Supplementary Material, Fig. S5), therefore the maximum in $I_4(T)$ cannot be related to local-scale transformations involving the existing non-tetragonal polar distortions. Thus, we assume that $I_4(T)$ mirrors the temperature evolution of the average of the non-tetragonal polar distortions, which may include a change in the magnitude and/or correlation length of the existing distortions as well as the nucleation of new distortions. Above 580 K the fractional intensity of P$_4$, $I_{n4} = \frac{I_4}{\sum _{i=1}^{4}(I_i)}$ , surpasses 0.5 (Fig. 3d, inset), indicating that the fraction of non-tetragonal displacements becomes dominant over the fraction of tetragonal distortions well below $T_C$. Moreover, both P$_4$ and P$_1$ persist well above $T_C$ (Fig. 3a and Fig. S6, Supplementary Material), that is, both tetragonal and non-tetragonal polar distortions exist in the paraelectric phase, which is in full accordance with a total neutron scattering analysis revealing non-zero polar displacements across $T_C$ and a high degree of local orientation disorder^17^. Both $I_{n4}(T)$ and $\omega _{4}(T)$ trends as well as the reversibility of the spectral changes on cooling down clearly demonstrate the key role of the non-tetragonal polar entities in the occurrence of ferroelectric-paraelectric phase transition in PT and emphasize the importance of order-disorder processes.

Thus, the Raman data presented here and the previously reported results from pair-distribution-function analysis^17^ are consistent with the model of Cho et al.^23^, according to which the non-tetragonal polar displacements confined around crystal-lattice defects influence the behavior of the whole system above a certain critical temperature corresponding to a critical correlation length. However, it should be mentioned that an increase in the Raman intensity might reflect not only an increase in the correlation length, but also an increase in the magnitude of the individual polar distortions and/or further nucleation of non-polar distortions.

We now consider the effects of substitutions. Similar to PT, the higher-energy subpeaks in the spectra of both PT-0.08BZT and PT-0.17BMT are suppressed with increasing temperature, while P$_4$ is enhanced (Fig. 2a and Fig. S6, Supplementary Material) and begins to dominate over P$_1$–P$_3$ already below $T_C$ (see the insets in Fig. 3e and f). However, PT-0.08BZT and PT-0.17BMT significantly differ from each other in the temperature dependences of $\omega _4$ and $I_4$. For PT-0.08BZT, $\omega _4(T)$ shows a typical soft-mode behavior (Fig. 3b) and a power-function fit reveals $\omega _{4}(T) \propto (805-T)^{0.29\pm 0.02}$. The *n* value suggests that the BZT substitution changes the character of developing non-tetrahedral distortions from second-order (PT) towards tricritical (PT-0.08BZT)^40,41^. Moreover, for PT-0.08BZT $I_4(T)$ shows a clear maximum at a temperature that is $\sim$85 K below $T_C$ (Fig. 3e), as in the case of PT, and the FWHM of the asymmetric Sigmoidal function fitting the $I_4(T)$ data points is even smaller than that for PT. For PT-0.17BMT (Fig. 3c), the softening rate of the lowest-energy subpeak is less pronounced compared to the other solid solutions, which suggests a significant contribution of the order-disorder phenomena across $T_C$ for PT-0.17BMT and is consistent with the results from a second-harmonic-generation analysis on a similar composition^43^. Furthermore, I$_4$ shows a maximum around 400 K (Fig. 3f), followed by a gradual decrease over a broad temperature range. Within this temperature range, the intensity ratios of the two peaks are comparable, suggesting an even fraction of tetragonal and non-tetragonal polar distortions.

We assume that the main reason for the different temperature behavior of the two solid solution compounds is the character of the B-O interactions: both Ti$^{4+}$ and Zn$^{2+}$ have a covalent bonding with oxygen through the hybridization of the Ti 3*d* orbitals with the O 2*p* orbitals and the hybridization of the Zn 4*s* and 4*p* orbitals with the 2*p* orbitals of oxygen, respectively, whereas the interaction between Mg$^{2+}$ and oxygen anions is predominantly ionic^44,45^. Thus, Mg$^{2+}$ cations act as “modifiers” of the BO$_6$-network connectivity, disturbing the correlation between the tetrahedrally distorted TiO$_6$ octahedra. This in turn indirectly promotes the non-tetragonal local distortions of the A-BO$_3$ species in a wide temperature range and enhances the order-disorder character of the phase transition.

**Figure 4:**
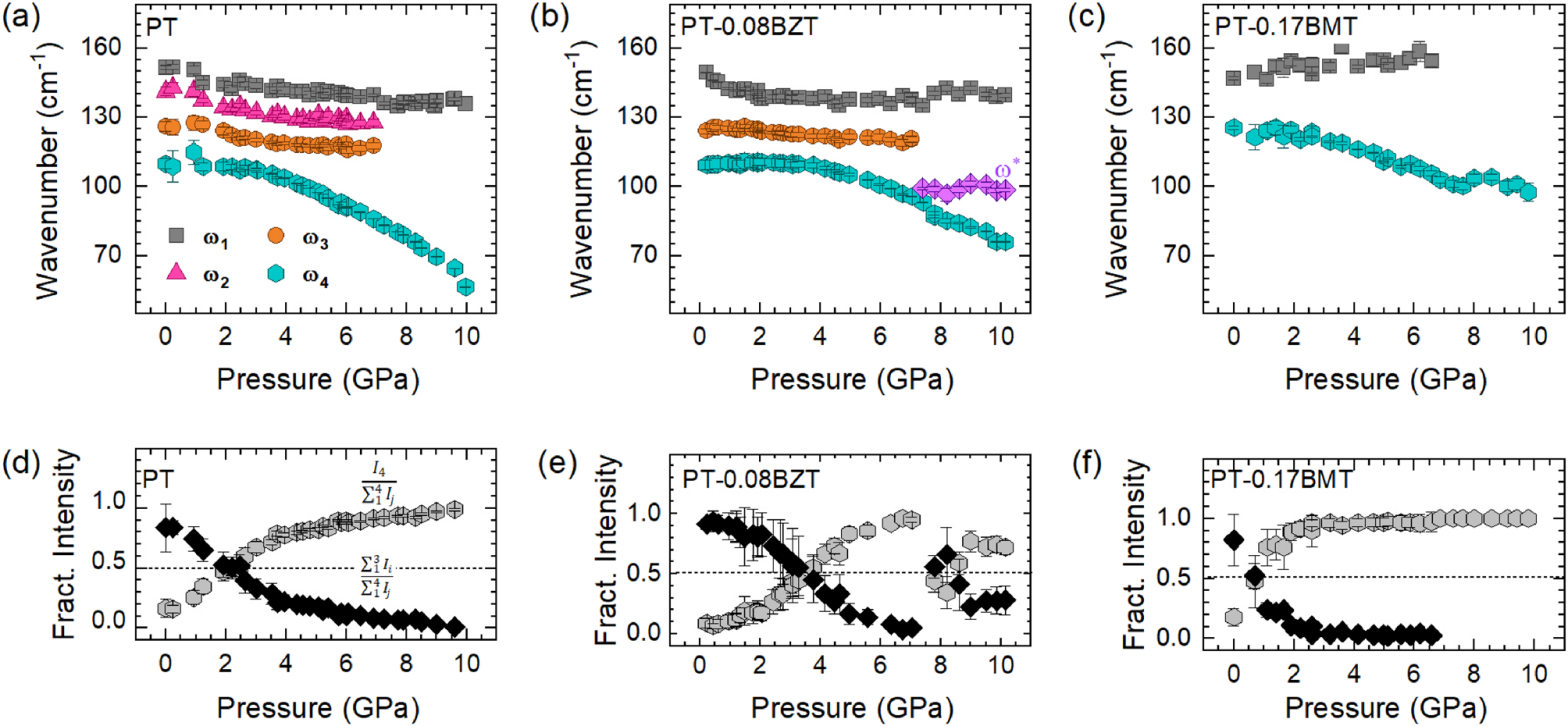
(**a**–**c**) Wavenumber $\omega$ of the A$_1$(1TO) phonon-subpeaks as a function of pressure and composition. (**d**–**f**) Intensity ratios $\frac{I_1+I_2+I_3}{I_1+I_2+I_3+I_4}$ (black symbols) and $\frac{I_4}{I_1+I_2+I_3+I_4}$ (gray symbols) for PT and PT-0.17BMT, $\frac{I_1+I_2+I_3+P*}{I_1+I_2+I_3+I_4+P*}$ and $\frac{I_4}{I_1+I_2+I_3+I_4+P*}$ for PT-0.08BZT.

Figure 2b shows that P$_4$ is also significantly enhanced under hydrostatic pressure, whereas P$_1$–P$_3$ are gradually suppressed. This indicates that pressure, like temperature, suppresses the polar tetragonal distortions and enhances the preexisting local polar non-tetragonal distortions, which is consistent with the previously reported *P*4*mm*-to-*R*3*c* phase transition of PT^12^. So far, the mixed compounds have not been analyzed by in situ high-pressure XRD.

Figure 4a–c show the pressure dependences of the wavenumber $\omega$ of the A$_1$(1TO)-mode subpeaks of the studied compounds. For PT (Fig. 4a), a slight decrease in the peak positions of the higher-energy subpeaks is observed up to 2 GPa. Upon further pressure increase, $\omega _1$–$\omega _3$ remain nearly constant. In contrast, $\omega _4(p)$ clearly softens upon compression. Several factors could account for the constant value of the wavenumber with increasing pressure: (1) the phonon compressibility is close to zero^46^, (2) the increase in $\omega$ due to the shrinking atomic distances is compensated by a mode softening at the same rate, or (3) the anharmonicity of the potential does not change with pressure in the corresponding range (for P$_2$ and P$_3$). A similar trend can be observed for PT-0.08BZT (Fig. 4b). Although the analysis of the pressure evolution of the peaks is complicated by the overlapping of P$_1$ and P$_2$, it is apparent that the wavenumbers of P$_1$+P$_2$ and P$_3$ are not sensitive to changes in pressure. Above 7.4 GPa an additional peak near 99 cm$^{-1}$, labeled P$^*$ in Fig. 2b, can be resolved for PT-0.08BZT (see Fig. S4 in the Supplementary Material for more details on the fitting procedure). Since the wavenumber of P$^*$ remains nearly constant with pressure, as P$_1$, P$_2$, and P$_3$ do for both PT and PT-0.08BZT, we assume that P$^*$ also originates from the anharmonicity of the interatomic potential, and it could not be observed at lower pressures as a result of overlapping with P$_4$. For PT-0.17BMT (Fig. 4c), the wavenumber of the higher-energy peak hardens on compression, whereas the wavenumber of the lower energy peak decreases almost linearly with the increase in pressure. The composition dependence of $\omega (p)$ indicates that the rate of softening decreases through increasing the concentration of the substitution.

The pressure dependencies of the fractional intensity $I_{n4} = \frac{I_4}{\sum _{i=1}^{4}(I_i)}$ (Fig. 4d–f) reveal that the non-tetragonal distortions prevail over the polar tetragonal distortion above 2.2 GPa for PT, 3.8 GPa for PT-0.08BZT, which suggests that B-site Zn$^{2+}$ slows down the progressive development of non-tetragonal distortions under pressure. For PT-0.17BMT, it is apparent that B-site Mg$^{2+}$ favors the non-tetragonal distortions already at relatively low pressures. However, the peak broadness and possible overlapping hinders the precise determination of the pressure above which the non-tetragonal distortions become dominant. Due to potential artifacts related to the depolarization of the diamond anvils, the pressure dependence of the absolute intensity $I_4$ (Supplementary Material, Figure S7) will not be discussed in detail.

## Conclusions

In conclusion, we demonstrate that above a certain characteristic temperature or pressure, the *atomic dynamics* of polar A-BO$_3$ entities distorted in a non-tetragonal way become dominant, even though the symmetry of the average structure is still polar tetragonal. Given that the A$_1$-mode polarization is along the tetragonal [001] direction (see Fig. 1), the “defect” A-type excitations violate the polar tetragonal long-range order along the direction of the macroscopic polarization. Consequently, these excitations significantly contribute to the temperature-/pressure-induced phase transitions of pure PT and its solid solutions with complex perovskites. Moreover, we show that Zn improves the resistivity of the polar tetragonal distortions to increasing pressure or temperature, whereas Mg favors the expansion of non-tetragonal distortions. This might explain the decrease in the unit-cell tetragonality of PbTiO$_3$-*x*Bi(Mg$_{0.5}$Ti$_{0.5}$)O$_3$ with increasing *x* and the existence of a tetragonal-to-rhombohedral composition-induced phase transition *P*4*mm* ${\mathop {\rightarrow }\limits ^{x \ = \ 0.63}} R3c$^9^. In contrast, the substitution of Bi(Zn$_{0.5}$Ti$_{0.5}$)O$_3$ only enhances the tetragonality of the unit cell^34^, without leading to a composition-driven phase transition.

## Methods

Single crystals of PbTiO$_3$, 0.92PbTiO$_3-$0.08Bi(Zn$_{0.5}$Ti$_{0.5}$)O$_3$, and 0.83PbTiO$_3-$0.17Bi(Mg$_{0.5}$Ti$_{0.5}$)O$_3$ were grown using the flux method and the top-cooled solution growth method^4,47^. The chemical composition and homogeneity of the samples were verified by wavelength-dispersive spectroscopy using a Cameca SX100 electron microprobe. Polarized Raman spectra were collected in the $\bar{x}(yy)x$ backscattering geometry with a Horiba Jobin-Yvon T64000 triple-grating spectrometer, using the 514.532-nm line of an Ar$^+$ laser. The spectral resolution was 2 cm$^{-1}$ and the peak position accuracy was $\sim$0.35 cm$^{-1}$. A detailed description of the experimental conditions and data evaluation is given in the Supplementary Material.

### Supplementary Information

Supplementary Information.

## Data Availability

All data analysed during this study are included in this published article and its Supplementary Material.
