# Supplementary material for: The role of local non-tetragonal polar displacements in the temperature- and pressure-induced phase transitions in PbTiO3-BiMeO3 ferroelectrics
Source: Sci Rep. 2024 Mar 26;14:7106. doi: 10.1038/s41598-024-57765-w (PMC11349899; doi:10.1038/s41598-024-57765-w)
Supplement: Supplementary file 1 — Supplementary Information. [file 41598_2024_57765_MOESM1_ESM.pdf]

The role of local non-tetragonal polar displacements in the temperature- and pressure-induced phase transitions in $PbTiO_3$-Bi*Me*O_3 ferroelectrics

Irina Margaritescu,[1] Zenghui Liu,[2] Zuo-Guang Ye,[3] and Boriana Mihailova[1]

[1]Department of Earth Sciences, Universität Hamburg, Grindelallee 48, 20146 Hamburg, Germany
[2]Electronic Materials Research Laboratory, Key Laboratory of the Ministry of Education & International Center for Dielectric Research, School of Electronic Science and Engineering, Xi'an Jiaotong University, Xi'an, 710049, China.
[3]Department of Chemistry and 4D LABS, Simon Fraser University, Burnaby, British Columbia V5A 1S6, Canada

Supplementary Material

Experimental details

Polarized Raman spectra were collected in the $\bar{x}(yy)x$ scattering geometry (*x* and *y* parallel to the tetragonal [100] and [010] directions, respectively) with a Horiba Jobin-Yvon T64000 triple-grating spectrometer equipped with an Olympus BH41 microscope. The 514.532-nm line of an Ar^+-laser was focused on the sample surface through a 50× long-working distance objective to a spot with a diameter of ~2 µm. The spectrometer was calibrated to the Raman peak at 520.5 cm^{-1} of a Si wafer. The spectral resolution was 2 cm^{-1} and the accuracy of the peak position was ~0.35 cm^{-1}.
The temperature-dependent measurements were carried out on (100)-oriented platelets with a size of ~1×1×0.5 mm^3, using a Linkam THMS-E600 stage on cooling between 300 K and 150 K and a Linkam TS1200 EV-1015 stage on heating between 300 K and 1000 K with a temperature step of 20 K.
The *in situ* high-pressure Raman measurements up to 10 GPa were conducted at room temperature on (100)-oriented platelets with a size of ~100×70×50 μm^3, using a Boehler-Almax diamond anvil cell (DAC) with diamond culets of 600 µm in diameter. Stainless steel gaskets were preindented to ~90 µm thickness and a gasket hole with a diameter of 300 µm was drilled using a spark eroder. A 16:3:1 methanol-ethanol-water mixture was used as a hydrostatic pressure transmitting medium and the R1 florescence line of a ruby crystal placed near the sample was used to determine the pressure. The spectra were collected with a pressure step of ~0.3 GPa and a pressure accuracy of ~0.1 GPa.

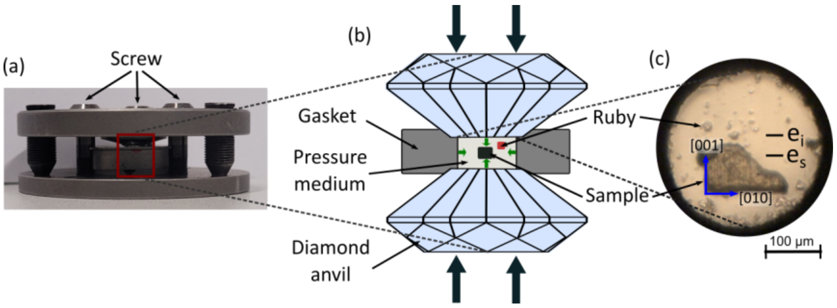

Figure S1: Graphical representation of the experimental setup used for the high-pressure Raman measurements (a) Lateral view of the diamond anvil cell (b) Schematic view of the diamond anvil cell. By simultaneously tightening the three pressure screws seen in (a) using a gear tool, the diamond anvils apply uniaxial pressure (gray arrows) to the gasket. The pressure medium transforms the uniaxial stress into hydrostatic pressure (green arrows). The pressure transmitting medium used in this study, 16:3:1 methanol-ethanol-water, ensures hydrostatic conditions up to 10.4 GPa[1]. (c) Sample and ruby crystal in the high-pressure chamber viewed from above. The direction of propagation of the incident and scattered light is along [100]. The polarization direction of the incident and scattered light (**e**$_i$ and **e**$_s$, respectively) is parallel to [010].

Fitting details

The experimental data were evaluated using the OriginPro 2019b software package[2]. To eliminate the trivial temperature dependence of the peak intensities due to the alteration in energy-level population, the measured Raman spectra were first corrected by the Bose-Einstein phonon occupation factor $I = I_{measured} \,/\, \left[\left(e^{\hbar\omega/k_BT} - 1\right)^{-1} + 1\right]$, where $\hbar$, ω, k_B, and T are the reduced Planck constant, phonon wavenumber (in s^{-1}), Boltzmann constant, and temperature, respectively. The temperature-reduced spectra were fitted with pseudo-Voigt functions PV = μL + $(1-\mu)G$, where L and G are Lorentzian and Gaussian peak-shape functions of equal width and μ is a weight coefficient. Thus for each peak four parameters were refined: the peak positions ω, full widths at half maximum (FWHMs) Γ, integrated intensities I and coefficients μ, where μ varies between 0 and 1. μ =1 corresponds to a pure Lorentzian peak-shape function, which is the natural peak shape of any damped harmonic oscillator, whereas $\mu < 1$ represents the Gaussian contribution to the peak shape related to the statistical dispersion over phonon energy, which can be inspected in materials with structural and/or compositional disorder[3]. It should be emphasized that Lorentzian or Voigtian peak-shape functions are routinely applied to ferroelectric materials[4-15], including solid solutions. An asymmetric peak-shape function related to the chemically induced folding of the Brillouin zone (e.g. Bergmann et al.[16]) is not necessary to be introduced for perovskite-type ferroelectrics (as the materials studied here), because they are wide-bandgap insulators and such effects are negligible[8].

The fittings were performed without putting any constraints on the variable parameters, apart from the μ coefficients, which were initially released to vary between 0 and 1 and then fixed to the resultant value only at the final step of fitting, after reaching the final values of all fitting parameters. In the case of PT the μ coefficients converged to 1, corresponding to a Lorentzian peak-shape function, whereas for PT-0.08BZT and PT-0.17BMT, the μ values varied in the range [0, 1]. In order to check the effect of the peak shape we have tested different fitting models, using pure Lorentzians, pure Gaussians or pseudo-Voigt functions with intermediate μ values and observed that the temperature and pressure trends of ω, I and Γ remain consistent regardless of the fitting peak-shape functions chosen.

The number of peak functions used to fit the spectra was based on the group-theory selection rules about the number of fundamental Raman-active phonon modes allowed to be observed in a certain scattering geometry. When the number of peaks deviated from the predictions of group theory, the number of used pseudo-Voigt peaks at each pressure or temperature step was justified by the standard criteria for goodness-of-fit: adjusted R^2, reduced χ^2, the relative uncertainties of each fitted parameter as well as the statistical t and prob>|t| values[7]. The t and prob>|t| values describe the fitted value divided by the error and the probability of a certain parameter to exist, respectively. The decision to keep or exclude a peak was based on the requirement the relative error in intensity $\Delta I/I$ to be smaller than 0.5 and the corresponding prob>|t| value to be smaller than 0.05. According to the null-hypothesis significance testing, a prob>|t| value below 0.05 supports the presence of the parameter, whereas a value higher than 0.05 indicates that the parameter should be removed. In the case where an additional peak was required, we have tried several fitting models while considering the R^2, reduced χ^2 and prob>|t| values. This procedure is shown below for PT-0.08BZT.

Group-theory analysis

The Γ-point optical phonon modes in the cubic $Pm\overline{3}m$ ABO_3 structure are Γ_{optic} = $3T_{1u} + T_{2u}$. In the tetragonal *P4mm* phase, each T_{1u} mode splits as A_1+E, whereas the T_{2u} mode splits as B_1+E. Furthermore, long-range electrostatic forces further split the polar A_1 and E modes into TO (transverse) and LO (longitudinal) components [13]. The phonon modes in the cubic phase are not Raman active, whereas the phonon modes in the tetragonal phase are all Raman active.
According to group-theory analysis, the E(TO), A_1(TO) + B_1, and A_1(LO) + B_1 modes can be observed in the $\bar{x}(zy)x$, $\bar{x}(yy)x$, and $z(xx)z$ scattering geometries, respectively.
The peak assignment for pure $PbTiO_3$ is in a good agreement with previous studies [14, 15].

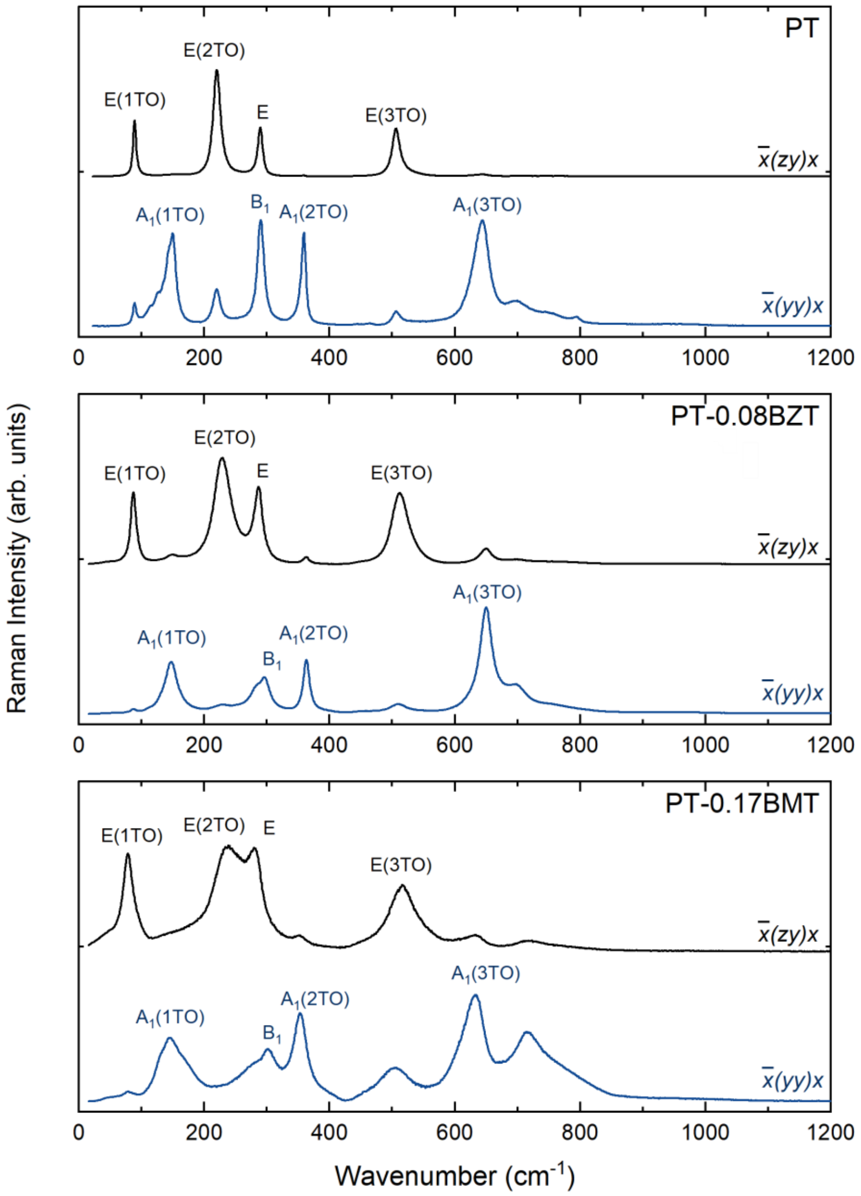

Figure S2: Raman spectra of $PbTiO_3$, PT-0.08BZT and PT-0.17BMT measured under ambient conditions in the $\bar{x}(zy)x$ and $\bar{x}(yy)x$ scattering geometries. Small intensity leakage of phonon modes in forbidden scattering geometries is due to unavoidable experimental imperfections.

Chemical analysis

The chemical composition and homogeneity of the mixed samples were determined by wavelength-dispersive electron microprobe analysis. Sections with a thickness of ~200 µm were cut from each sample and mounted in an epoxy disk. The experiments were carried out with a Cameca Microbeam SX100 equipped with a wavelength-dispersive detector. An accelerating voltage of 15 keV and a beam current of 20 nA were used. The beam diameter on the sample surface was ~10 µm. The following standards were used for the calibration: MgO for Mg, TiO_2 for Ti, complex silicate glass for Pb and Zn, and $Bi_4Ge_3O_{12}$ for Bi. The chemical composition of each sample was obtained by averaging over 60-130 spatial points collected along several lines set across the crystals. The obtained chemical compositions are given in Table S1.

	Pb^{2+}	Bi^{3+}	Ti^{4+}	Zn^{2+}	Mg^{2+}	O^{2-}	Chemical formula
PT-BZT	17.40(15)	1.66(6)	19.54(11)	0.93(4)	-	60.33(5)	$0.92PbTiO_3$-$0.08Bi(Zn_{0.5}Ti_{0.5})O_3$
PT-BMT	15.85(14)	3.38(8)	18.60(11)	-	1.71(5)	60.28(6)	$0.83PbTiO_3$-$0.17Bi(Mg_{0.5}Ti_{0.5})O_3$

Table S1: Chemical composition in atomic percentage and chemical formulae of the studied samples. The statistical standard deviations are given in brackets.

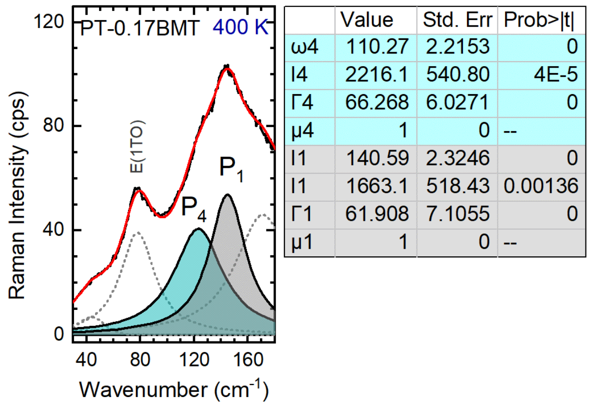

	Value	Std. Err	Prob>|t|
ω4	110.27	2.2153	0
I4	2216.1	540.80	4E-5
Γ4	66.268	6.0271	0
μ4	1	0	--
I1	140.59	2.3246	0
I1	1663.1	518.43	0.00136
Γ1	61.908	7.1055	0
μ1	1	0	--

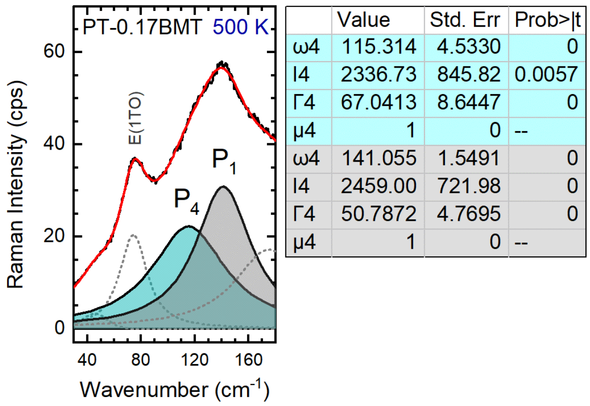

	Value	Std. Err	Prob>|t
ω4	115.314	4.5330	0
I4	2336.73	845.82	0.0057
Γ4	67.0413	8.6447	0
μ4	1	0	--
ω4	141.055	1.5491	0
I4	2459.00	721.98	0
Γ4	50.7872	4.7695	0
μ4	1	0	--

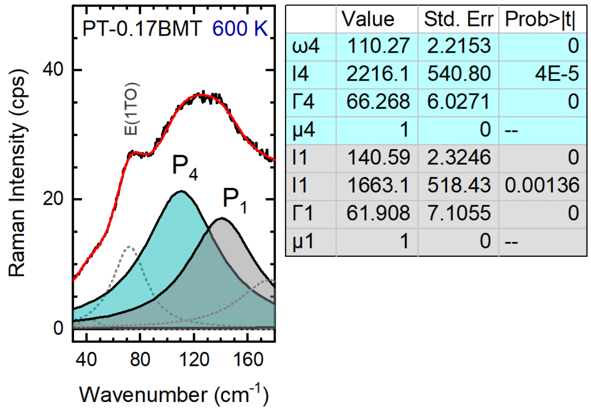

	Value	Std. Err	Prob>|t|
ω4	110.27	2.2153	0
I4	2216.1	540.80	4E-5
Γ4	66.268	6.0271	0
μ4	1	0	--
I1	140.59	2.3246	0
I1	1663.1	518.43	0.00136
Γ1	61.908	7.1055	0
μ1	1	0	--

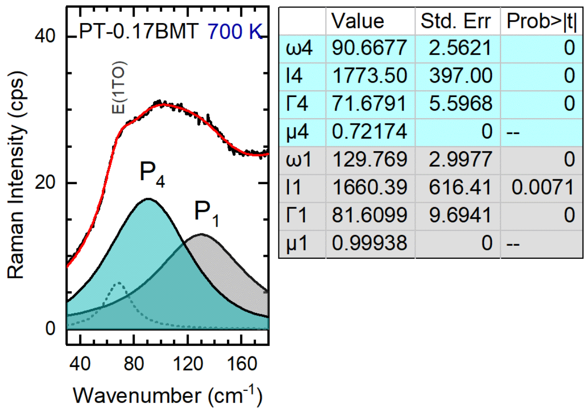

	Value	Std. Err	Prob>|t|
ω4	90.6677	2.5621	0
I4	1773.50	397.00	0
Γ4	71.6791	5.5968	0
μ4	0.72174	0	--
ω1	129.769	2.9977	0
I1	1660.39	616.41	0.0071
Γ1	81.6099	9.6941	0
μ1	0.99938	0	--

Figure S3: Raman spectra of PT-0.17BMT collected at 400K, 500 K, 600 K, and 700 K. The Raman peak positions ω, FWHMs Γ, integrated intensities I, and the Lorentzian contribution μ, along with their corresponding errors and prob>|t| values are shown in the tables next to the figures. The prob>|t| values of I_1 and I_4 are below 0.05, as required by the null-hypothesis significance testing, which indicates that two peaks should be used to fit this spectral region.

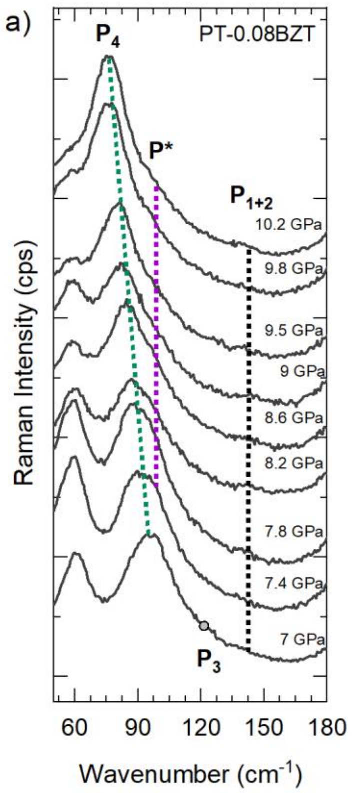

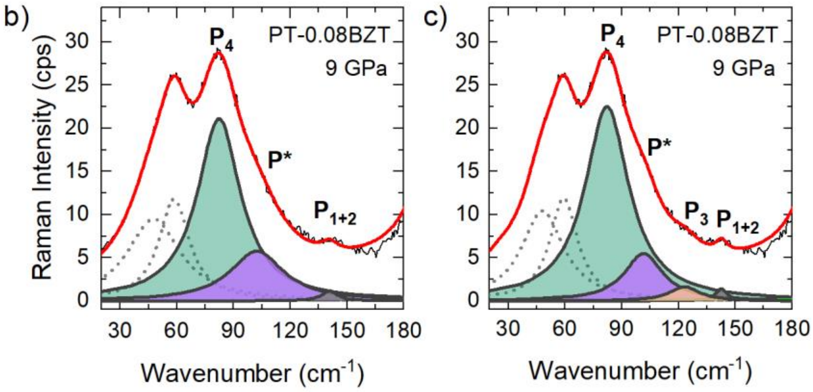

		Value	Std. Error	Prob>|t|
P_4	ω	81.95444	0.39208	0
	I	848.0038	128.11965	0
	Γ	27.13438	1.96542	0
	μ	1	0	--
P*	ω	100.5129	2.774	0
	I	353.8711	109.51372	0.00125
	Γ	37.81514	4.61554	0
	μ	0.8701	0	--
P_{1+2}	ω	141.0429	1.21292	0
	I	18.89372	5.91926	0.00144
	Γ	12.48042	3.92354	0.00149
	μ	0.67216	0	--

	Value	Std. Error	Prob>|t|	
ω	82.26889	0.41827	0	
I	910.5012	109.5072	0	
Γ	27.29606	1.75279	0	
μ	0.98302	0	--	
ω	101.3757	1.31661	0	
I	209.9004	93.6888	0.02518	
Γ	25.25074	6.49373	0	
μ	0.88649	0	--	
ω	123.5710	2.04274	0	P_3
I	52.33609	34.90695	0.10396	
Γ	19.50017	9.23034	0.03477	
μ	0.98089	0	--	
ω	142.4837	0.99871	0	
I	19.04256	7.35835	0.00973	
Γ	10.09264	3.49444	0.00392	
μ	0.73088	0	--	

Figure S4**:** a) Raman spectra of PT-0.08BZT collected between 7 and 10.2 GPa. Above 7.4 GPa a new peak (P*) is observed; the dashed lines trace peaks P_{1+2} near 140 cm^{-1}, P_4 near 80 cm^{-1}, and P* near 100 cm^{-1}. Figures b) and c) show the fitting of the Raman spectra collected at 9 GPa using pseudo-Voigt functions and two different models with 3 (b) and 4 (c) peaks. The values of the Raman peak positions ω, FWHMs Γ, integrated intensities I, and the Lorentzian contribution μ, as well as the corresponding errors and prob>|t| values are given in the tables below the figures. In the pressure range 7.4-10.2 GPa, the prob>|t| value of I_3 (the intensity of peak P_3) starts oscillating and at some pressures it exceeds 0.05. For example, for P_3, at 9 GPa, $\Delta I/I > 0.5$ and prob>|t| ~ 0.1. Therefore, we have removed this weak peak from the fitting model in the pressure range 7.4-10.2 GPa.

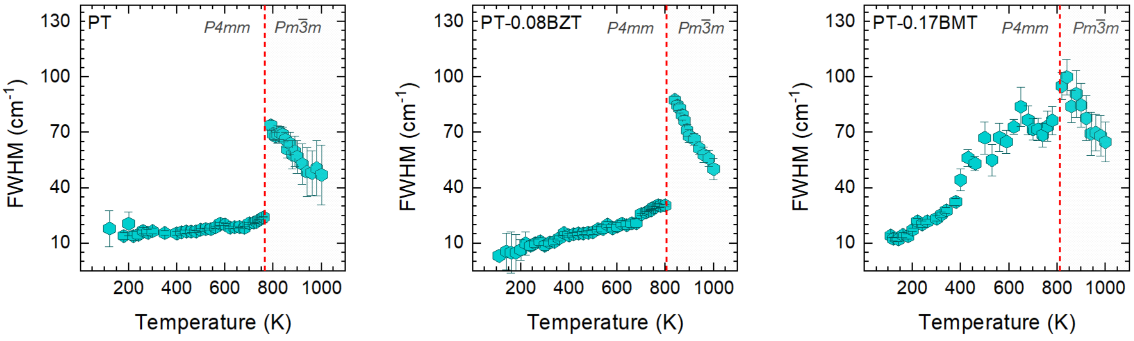

***Figure S5**:* Full width at half maximum *(*FWHM) of the lowest energy subpeak P_4 as a function of temperature for PT, PT-0.08BZT and PT-0.17BMT.

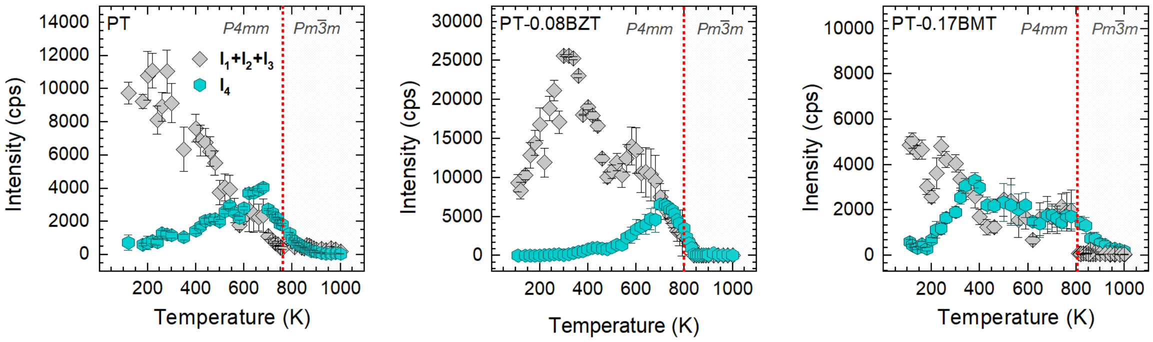

***Figure S6**:* Raman intensity of the lowest-energy subpeak P_4 and sum of the Raman intensities of the highest-energy subpeaks P_1-P_3 as a function of temperature for PT, PT-0.08BZT and PT-0.17BMT.

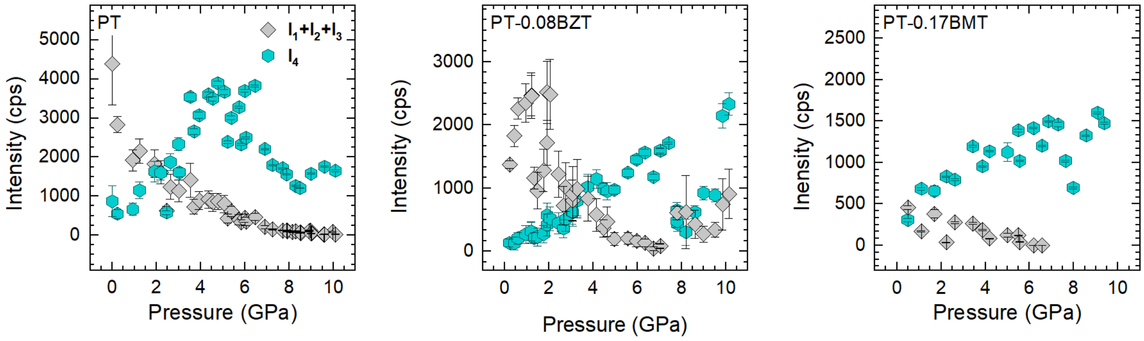

Figure S7: Raman intensity of the lowest-energy subpeak P_4 and sum of the Raman intensities of the highest-energy subpeaks P_1-P_3 as a function of pressure for PT, PT-0.08BZT and PT-0.17BMT.

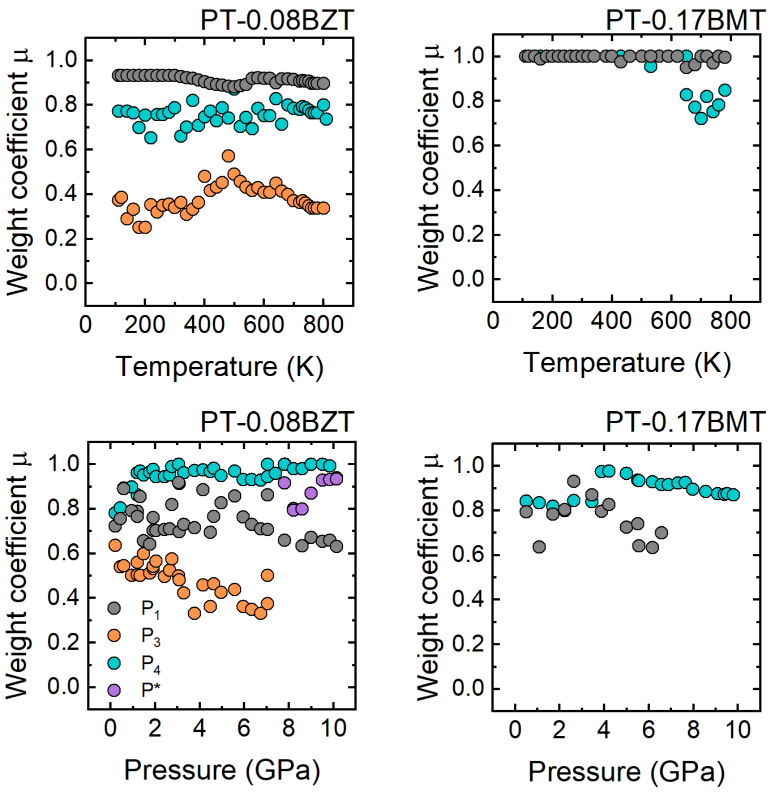

Figure S8: Weight coefficient μ as a function of temperature and pressure for PT-0.08BZT and PT-0.17BMT. For pure PT, unconstrained fits yielded weight coefficients ~1, and therefore pure Lorentzian peak shape functions were used in the final fits.

References

[1] R. J. Angel, M. Bujak, J. Zhao, G. D. Gatta, and S. D. Jacobsen, J. Appl. Crystallogr. 40, 26 (2007).
[2] OriginPro, Version 2019b. OriginLab Corporation, Northampton, MA, USA.
[3] H. Kuzmany, Solid State Spectroscopy, Springer, 1998, page 29.
[4] C. M. Foster, Z. Li, M. Grimsditch, S.-K. Chan, and D. J. Lam, Phys. Rev. B 48, 10160 (1993).
[5] S. M. Cho, H. M. Jang, and T.-Y. Kim, Phys. Rev. B 64, 014103 (2001).
[6] J. Toulouse, F. Jiang, and O. Svitelskiy, Phys. Rev. B 72, 184106 (2005).
[7] U. Bismayer, V. Devarajan and P. Groves, J. Phys.: Condens. Matter 1, 6977 (1989).
[8] I. Margaritescu, K. Datta, J. Chen, and B. Mihailova, J. Raman Spectrosc. 51, 1200 (2020).
[9] J. Rouquette, J. Haines, V. Bornand, M. Pintard, Ph. Papet, and J. L. Sauvajol, Phys. Rev. B 73, 224118 (2006).
[10] J. Frantti; Y. Fujioka; A. Puretzky; Y. Xie; Z.-G. Ye; A. M. Glazer, J. Appl. Phys. 113, 174104 (2013).
[11] J. Hlinka, I. Gregora, and V. Vorlíček, Phys. Rev. B 65, 064308 (2002).
[12] V. Buscaglia et al., J. Phys.: Condens. Matter 26, 065901 (2014).
[13] G. Burns and B. A. Scott, Phys. Rev. B 7, 3088 (1973).
[14] C. M. Foster, Z. Li, M. Grimsditch, S.-K. Chan, and D. J. Lam, Phys. Rev. B 48, 10160 (1993).
[15] R. A. Frey and E. Silberman, Helv. Phys. Acta 49, 1 (1976).
